# The livestock drinking water system as an active reservoir for antimicrobial resistance: A systematic review and one health gap analysis

**DOI:** 10.1371/journal.pone.0349556

**Published:** 2026-06-03

**Authors:** Sujan Adhikari, Swagat Khanal, Akash Adhikari

**Affiliations:** 1 Faculty of Animal Science, Veterinary Science and Fisheries, Agriculture and Forestry University, Chitwan, Nepal; 2 Institute of Agriculture and Animal Science, Tribhuvan University, Bhairahawa, Nepal; The Hong Kong Polytechnic University, HONG KONG

## Abstract

Livestock drinking water distribution systems represent a critical but understudied interface in the epidemiology of antimicrobial resistance. While engineered for production, these systems frequently function as unintended bioreactors where biofilms protect pathogens and facilitate horizontal gene transfer. Following PRISMA and SWiM guidelines, we systematically searched four databases (MEDLINE, Scopus, AGRIS, PubAg) through November 2025 for primary research on antimicrobial resistance in livestock water biofilms. Eligible studies underwent risk-of-bias assessment using JBI tools. Due to substantial methodological heterogeneity in sampling and assays, data were synthesized narratively to characterize resistance prevalence and reservoir dynamics. The synthesis reveals that DWDS biofilms harbor distinct microbial communities compared to transient planktonic or fecal inputs. Critically, these matrices sustain critical priority traits, including multidrug efflux pumps (*adeF*) in swine systems, plasmid-mediated colistin (*mcr-1* to *mcr-5*) and carbapenemase (*blaNDM*) genes. Evidence indicates that standard disinfection protocols often fail to eliminate established biofilms, allowing rapid recolonization by resistant populations within days of treatment. These findings suggest that farm water infrastructure acts as a persistent reservoir for genetic resistance traits, capable of reseeding animal cohorts despite distinct production cycles. We identify a critical surveillance blind spot and conclude that current One Health surveillance strategies should expand beyond bulk water testing to include targeted biofilm sampling. Effective mitigation requires engineering solutions and enzymatic treatments specifically designed to disrupt the protective matrix, thereby closing a significant gap in on-farm biosecurity.

## Introduction

Antimicrobial resistance (AMR) is a defining challenge of the twenty-first century, undermining the effectiveness of clinical therapeutics and threatening animal health, food security, and ecosystem integrity [1–3]. Historically, agricultural AMR research has prioritized established reservoirs. The primary focus has been on antimicrobial usage, selection pressures in manure [4–6], and dissemination through the food chain. However, engineered on-farm infrastructures that sustain everyday production can themselves create ecological niches that foster microbial persistence and genetic exchange [7,8]. One such under examined niche is the farm drinking water distribution system (DWDS). This extensive infrastructure comprising pipes, tanks, and drinkers serves as a physical interface that connects animal, human, and environmental health.

In intensive livestock systems, DWDS operate under a set of conditions that are particularly permissive for microbial colonization. Low hydraulic shear, intermittent flow, warm temperatures, and the regular addition of organic supplements or medicated water create ideal conditions for biofilm development [9–12]. Biofilms are organized microbial communities held together by a protective matrix that changes local chemical conditions, alters microbial behavior, and shields the cells from chemical and physical stress [13,14]. These properties give biofilms a capacity for persistence distinct from that of transient planktonic bacteria [15,16] and make them important considerations for both routine hygiene and long-term control of microbial hazards in animal production systems [7].

Beyond persistence, biofilms have ecological attributes that are directly relevant to AMR dynamics. High cell densities and close physical proximity within the extracellular matrix increase opportunities for horizontal gene transfer (HGT), including conjugation of plasmids, mobilization of transposons, and uptake of extracellular DNA [17–19]. Physiological heterogeneity within biofilms, characterized by layers of actively growing, slow-growing, and dormant cells, gives rise to subpopulations referred to as persisters [20]. These cells exhibit elevated tolerance to antimicrobial agents and can subsequently repopulate the biofilm community following sanitization [21,22]. Finally, biofilms readily colonize a variety of pipe materials and micro-topographies and can embed within mineral scale or organic deposits that further shield them from disinfectants [23–25]. Together, these features mean that DWDS biofilms can act as both a refuge for antimicrobial-resistant bacteria and a hotspot for the maintenance and dissemination of resistance determinants.

Despite these mechanistic reasons to be concerned, surveillance and control strategies in livestock production have rarely prioritized DWDS biofilms. No standardized international guidelines exist for sampling the internal surfaces of farm water pipes, effectively leaving this reservoir unmonitored. Standard monitoring practices largely focus on bulk water quality metrics, animal sampling, and manure testing [26,27]. Even when water systems are assessed, focus is typically directed toward free-floating microorganisms or indicator counts rather than the surface-associated communities that dominate these systems [7,28,29]. This gap is consequential because biofilm-associated microbes can differ taxonomically and functionally from planktonic populations, and because biofilm reservoirs are less responsive to routine cleaning regimes designed for bulk water [30]. Moreover, the DWDS serves as a critical link among animals, caretakers, and the surrounding environment. Contaminated lines can repeatedly expose successive cohorts of animals, generate aerosols during cleaning that may reach workers, and release biofilm fragments along with associated genetic material into on-farm effluents and nearby ecosystems [9,31–33].

The emergent literature that explicitly samples biofilms from livestock water systems has begun to document both the diversity of biofilm microbiomes [34] and the presence of antimicrobial resistance genes (ARGs) within these communities [35,36]. However, studies are geographically scattered, employ a variety of sampling and analytical methods, and differ in their reporting of outcomes, making synthesis difficult. At the same time, practical questions remain unanswered: how common and persistent are clinically relevant ARGs in DWDS biofilms across production systems? Which engineering and management factors most strongly predict biofilm accumulation and ARG abundance? And critically, what gaps in surveillance and intervention design prevent effective incorporation of DWDS biofilms into One Health AMR mitigation strategies?

This review addresses these questions by systematically identifying and synthesizing primary research that explicitly samples biofilms from livestock DWDS and reports antimicrobial resistance outcomes. Our aim is to characterize the state of evidence on the prevalence, composition, and persistence of biofilm-associated antimicrobial resistance in farm water systems; to identify recurring infrastructure and management drivers; and to highlight methodological and knowledge gaps that deserve priority in a One Health research and policy agenda.

## Materials and methods

### Protocol, reporting, and registration

This systematic review was conducted according to a pre-specified protocol and is reported in conformity with the Preferred Reporting Items for Systematic Reviews and Meta-Analyses (PRISMA) 2020 statement [37]. Where quantitative synthesis was not appropriate owing to heterogeneity in study designs and outcomes, reporting and narrative synthesis followed the Synthesis Without Meta-analysis (SWiM) guidance [38]. The review protocol was prospectively registered with OSF Registries (Registration https://doi.org/10.17605/OSF.IO/NKF7X). The searches covered database inception through 15 November 2025. The completed PRISMA 2020 checklist is available in S1 Checklist.

### Ethics approval and consent to participate

Not applicable.

### Eligibility criteria

We included primary empirical studies that sampled biofilm material directly from farm DWDS, encompassing infrastructure such as pipes, nipple lines, drinkers, troughs, tanks, or detachable coupons. To be eligible, investigations were required to report at least one specific outcome measured within the biofilm matrix. These outcomes included phenotypic antimicrobial susceptibility determined via culture and susceptibility testing, or the molecular detection and quantification of antimicrobial resistance genes using techniques such as PCR, qPCR, targeted assays, 16S-based resistome inference, whole-genome sequencing, or shotgun metagenomics. Studies reporting antimicrobial residue measurements in biofilms by validated analytical methods, such as mass spectrometry, were also retained. The review covered poultry, cattle, swine, and small ruminant systems in both commercial and smallholder settings. To minimize publication bias, no restrictions were applied regarding the language of publication, analytical platform or publication year.

We excluded reviews, editorials, and conference abstracts lacking primary data. Studies restricted to bulk water, soil, manure, or wastewater without paired biofilm data were ineligible. In vitro models were excluded unless they explicitly simulated farm conditions using field-derived isolates. Investigations of municipal or wildlife water systems were excluded if they lacked a direct connection to livestock distribution infrastructure.

### Information sources and search strategy

We searched the following bibliographic and thesis databases (coverage: inception to 15 December 2025): MEDLINE (via PubMed), Scopus (Elsevier), AGRIS (FAO), and PubAg (USDA). Google Scholar was searched (exporting the first 200 results per query via Publish or Perish software [39], as well as ProQuest Dissertations & Theses.

A draft search concept was adapted for each database. The core strategy combined terms related to biofilms, drinking water systems on livestock farms, target animal species, and antimicrobial resistance. Full database-specific search strings are provided in S1 File.

### Study selection

All search results were exported to Zotero 7.0.16 (www.zotero.org) for deduplication using the internal duplicate-finder followed by manual verification. Screening proceeded in two stages. For title and abstract screening, two independent reviewers screened records using a standardized checklist (S1 Table). Decisions were recorded as ‘Include’, ‘Maybe’, or ‘Exclude’. Studies categorized as ‘Include’ and ‘Maybe’ were processed for full text screening.

**Fig 1:**
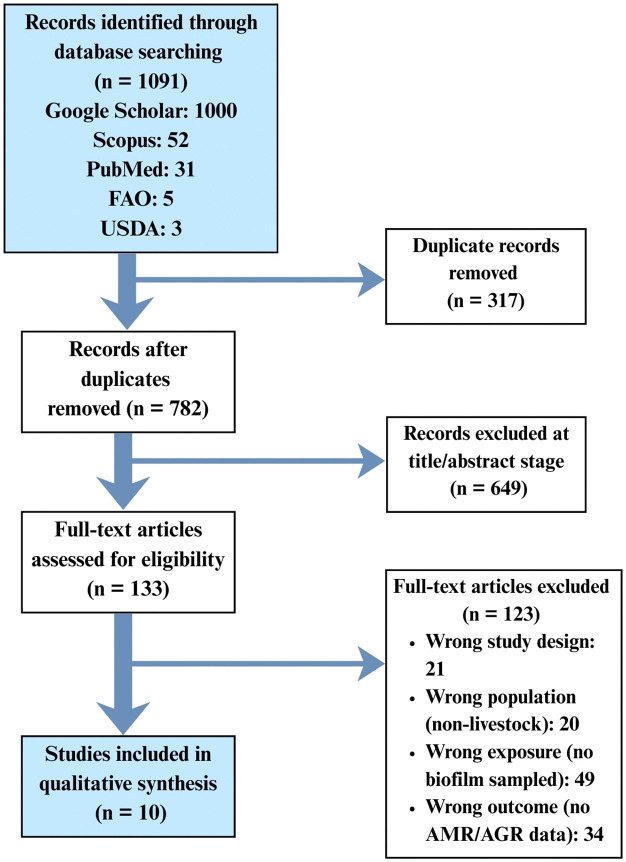
PRISMA 2020 flow diagram depicting the selection process of studies included in the systematic review.

Full texts were retrieved for potentially eligible records and screened independently by two reviewers. Disagreements were resolved by discussion or adjudication by a third senior reviewer. Reasons for exclusion at the full-text stage were coded and are reported in the PRISMA flow diagram (Fig 1).

### Risk of bias assessment

Methodological quality was assessed using an adapted risk-of-bias tool informed by the Joanna Briggs Institute (JBI) checklists and quality domains relevant to environmental microbiology [40]. Assessed domains included sampling representativeness, sample handling, laboratory controls, adherence to AST standards, molecular rigor, and reporting completeness. Two reviewers independently assigned a risk rating (Low, Moderate, High) to each study. Inter-rater reliability showed fair agreement of 66.7% and a Cohen’s κ of 0.38. Discrepancies were resolved through discussion to reach a consensus rating. Detailed domain-specific and overall ratings are provided in S2 Table.

### Data synthesis and analysis

Data management and descriptive analysis were performed using Microsoft Excel 2021 and R software version 4.4.3 (R Core Team, Vienna, Austria). A formal quantitative meta-analysis was not feasible due to the substantial heterogeneity observed across the included studies, particularly regarding sampling strategies and outcome metrics. Consequently, results were synthesized narratively according to the SWiM framework [38].

Studies were grouped primarily by the livestock production sector (poultry, swine, cattle) and secondarily by analytical platform (phenotypic versus genotypic) to facilitate logical comparison. Risk of bias ratings were utilized to characterize the strength of the evidence and discuss confidence in the findings, but were not used as criteria for exclusion. Findings were summarized using descriptive statistics, including frequencies and ranges. Graphical visualizations were generated using R and Canva (www.canva.com).

## Results

### Search results and study selection

The systematic search of bibliographic databases yielded a total of 1,091 records. After removing duplicates and screening titles/abstracts, full texts were retrieved for detailed assessment. A total of ten studies met the full eligibility criteria and were included in the review (Table 1). The selection process is detailed in the PRISMA flow diagram (Fig 1).

**Table 1:** Characteristics of included studies investigating DWDS biofilms in livestock.

Study (First Author, Year)	Country	Livestock Sector	Sampling Target	Laboratory Approach	Key Findings	References
Hayer, 2022	Germany	Dairy Cattle	Troughs (surfaces/biofilm)	Culture, ATP	*E. coli* in 48.6% of samples; MRSA and cephalosporin-resistant bacteria detected in biofilm.	[41]
Grudlewska-Buda, 2023	Poland	Swine	Troughs, Drinkers	Culture, PCR	VRE (*vanA, vanB*) detected; VRE strains showed significantly higher biofilm capacity.	[42]
Doughan, 2025	USA	Swine	PVC Waterline Coupons	Metagenomics, Culture	3,904 ARG hits (~184 unique genes); rapid regrowth of ARGs 3–7 days post-disinfection.	[43]
Piccirillo, 2024	Italy	Poultry (Broiler)	Pipe Biofilm	16S rRNA, qPCR	*bla*NDM and *mcr-5* detected; biofilm microbiome significantly different from feces.	[35]
Aboelseoud, 2021	Egypt	Poultry (Layer)	Iron & PVC Pipes	Culture, 16S rRNA	PVC pipes harbored significantly higher biofilm loads; 67% of isolates were MDR.	[44]
Heinemann, 2020	Germany	Poultry (Broiler)	Water lines, Sprinklers	Culture, MALDI-TOF	*P. aeruginosa* persisted in lines; resistant *Enterobacter* traced to hatchlings.	[45]
Ahangaran, 2022	Iran	Poultry (Broiler)	Water Biofilm	Culture, PCR	70% of *E. coli* tetracycline-resistant; 85.7% of resistant strains carried *tetA/tetB*.	[46]
Grakh, 2022	India	Poultry (Broiler)	Drinkers, Pipes	Culture, Vitek 2	91.5% of APEC were MDR; strong biofilm producers had 100% gentamicin resistance.	[47]
Vougat Ngom, 2025	Cameroon	Poultry (Mixed)	Water lines	qPCR, 16S rRNA	High prevalence of *mcr* and *bla*NDM; good AMU practices reduced polymyxin resistance.	[48]
Ren, 2025	China	Swine	SS Pipe Biofilm	Metagenomics	Microbiome diversity increased with pig age; *adeF* was the dominant ARG; *vanT* and *sul1* also detected.	[34]

### Characteristics of included studies

**Fig 2:**
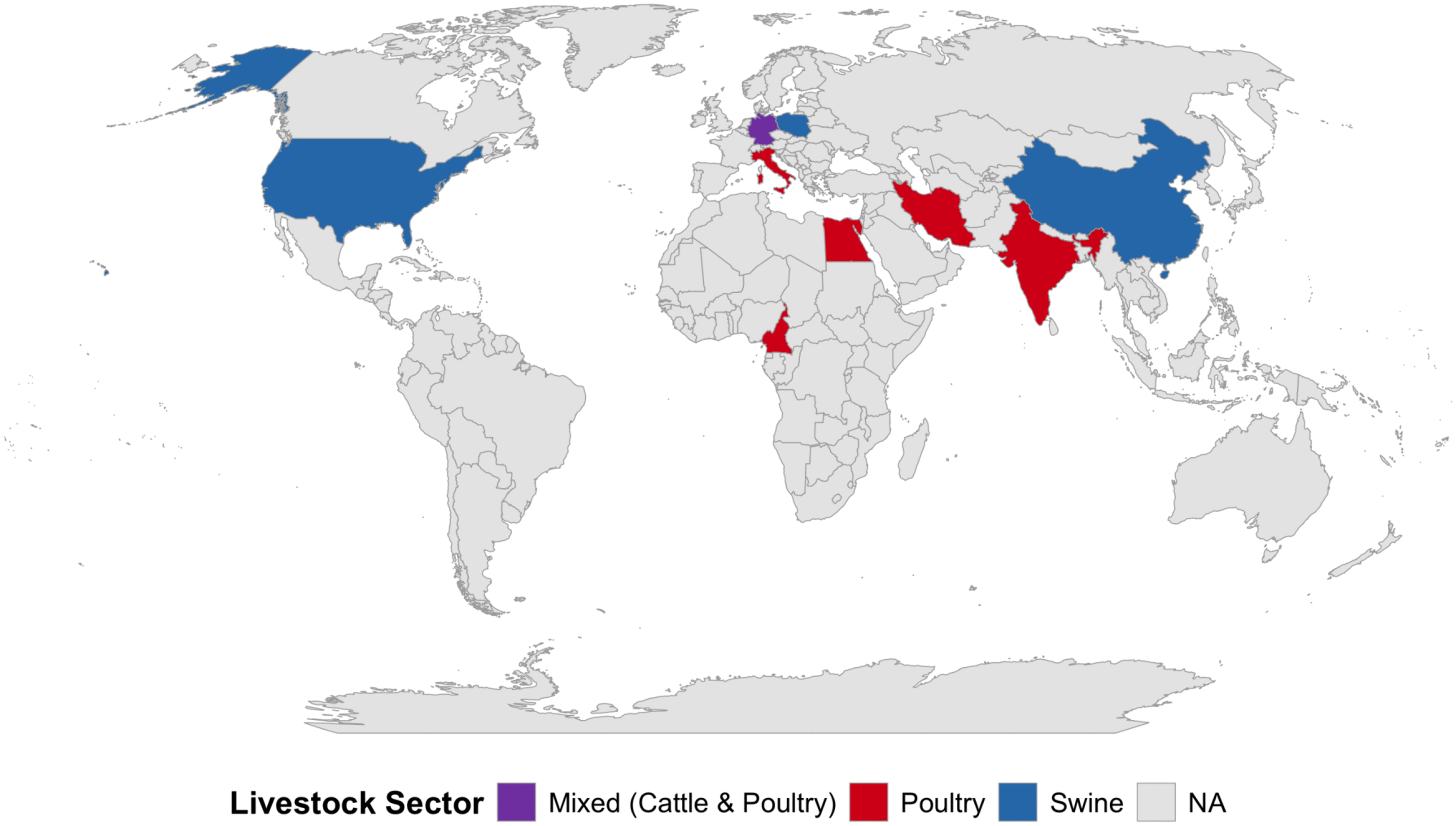
Global distribution of included studies (n = 10) by livestock sector. Base map data sourced from Natural Earth (public domain).

The included studies were published between 2020 and 2025, reflecting a recent emergence of interest in this niche. The ten eligible studies covered diverse geographies: Europe (n = 4), Africa (n = 2), Asia (n = 3), and North America (n = 1). However, significant data gaps remain in major production hubs like South America (Fig 2).

Poultry systems were the most frequently investigated sector (60.0%, n = 6), comprising broiler and layer farms. Three studies investigated swine facilities (Poland, USA, China), and one focused on dairy cattle troughs (Germany). Sampling strategies varied: destructive sampling of pipe sections or use of removable coupons was employed in three studies, while the remainder utilized swabbing of internal surfaces (pipes, drinkers, troughs). A summary of study characteristics is provided in Table 1.

### Methodological quality and risk of bias

Methodological quality was variable across the included datasets. Key limitations identified included: (1) lack of longitudinal sampling (only 3/10 studies sampled at multiple timepoints); (2) insufficient reporting of cleaning and disinfection history prior to sampling; and (3) variable reporting of AST standards. Only one study [43] utilized shotgun metagenomics to provide a comprehensive resistome profile; the remainder relied on culture-dependent isolates or targeted PCR/qPCR. Detailed risk of bias assessments are provided in S2 File.

### Microbial composition of farm DWDS biofilms

Analysis of microbial community structures indicated differences between biofilm residents and transient fecal microbiota. In Italian organic broiler farms, 16S rRNA sequencing identified *Proteobacteria* as the dominant phylum in water line biofilms, with high relative abundances of *Sphingomonadaceae* and *Burkholderiaceae.* These biofilm profiles were taxonomically distinct from the microbiomes of fecal samples collected from the same flocks [35]. This distinction was also observed in Cameroonian poultry systems, where biofilms harbored *Comamonadaceae* and other opportunistic pathogens that were not prevalent in paired fecal samples [48].

Regarding mammalian livestock, studies in swine production facilities recovered *Enterococcus* spp. and *Escherichia coli* directly from DWDS surfaces [42,43]. In dairy cattle settings, Hayer et al. (2022) detected *Pseudomonas* spp. and coliforms in water troughs, reporting that the recovery of these organisms varied significantly depending on the material composition of the trough surface [41].

In a metagenomic study of Chinese swine farms, Ren et al. [34] reported an age-related shift in biofilm communities within stainless steel water pipes. Biofilms in nursery barns were dominated by *Brevibacterium,* whereas those in finishing barns were mainly composed of *Brevundimonas* and *Sphingomonas.* Importantly, these biofilm communities shared several core taxa, including members of *Actinobacteria*, with porcine oral fluids, suggesting a bidirectional microbial exchange between pigs and the water distribution system.

### Phenotypic antimicrobial resistance

Phenotypic susceptibility testing was reported in seven studies, with multidrug resistance (MDR) frequently observed among biofilm-associated isolates. In Indian broiler farms, Grakh et al. (2022) reported that 91.5% of Avian Pathogenic E. coli (APEC) isolates were MDR, exhibiting specific resistance to enrofloxacin (89.4%) [47]. Similarly, investigation of layer farms in Egypt revealed that 67% of biofilm isolates were classified as MDR [44]. Regarding the association between biofilm formation and resistance phenotypes, Grudlewska-Buda et al. (2023) observed that Vancomycin-Resistant Enterococci (VRE) isolated from swine environments possessed significantly higher biofilm-forming capacity compared to susceptible strains [42]. This correlation was further supported by Grakh et al. (2022), who found that strong biofilm-producing E. coli demonstrated 100% resistance to gentamicin, a rate significantly higher than that observed in non-producing strains [47].

### Resistome and mobilization potential

Metagenomic and PCR evidence confirmed the presence of highest-priority critically important resistance genes (Table 2). Specifically, plasmid-mediated *mcr-1* through *mcr-5* and carbapenemase *blaNDM* genes were recovered from poultry water lines [35,48]. High-resolution shotgun metagenomics of swine water lines characterized the extensive breadth of the resistome, identifying 3,904 ARG hits representing 184 unique genes in US swine coupons [42]. Similarly, metagenomic profiling of Chinese swine farms identified 30 distinct ARGs in pipe wall biofilms. The multidrug efflux pump gene *adeF* exhibited the highest relative abundance, particularly in finishing barns, alongside vancomycin (*vanT*) and sulfonamide (*sul1*) resistance genes [34].

**Table 2:** High-priority antimicrobial resistance genes (ARGs) and phenotypes reported in farm DWDS biofilms.

Antimicrobial Class	Gene Targets/ Phenotypes Detected	Host Taxa (if identified)	References
Polymyxins	*mcr-1, mcr-2, mcr-3, mcr-4, mcr-5*	Community DNA	[35,48]
Carbapenems	*bla*NDM, *bla*VIM-2	Community DNA	[35,48]
Glycopeptides	*vanA, vanB, vanD*	*Enterococcus faecalis/faecium*	[42]
Tetracyclines	*tet(A), tet(B)*, Phenotypic resistance (up to 89%)	*E. coli*	[46,47]
Quinolones	*qnrS, oqxA, oqxB*; Phenotypic resistance (Enrofloxacin)	*E. coli*; Community DNA	[47,48]
Macrolides	*ermA, ermB*	Community DNA	[35,48]
Cephalosporins	Phenotypic resistance (3rd gen); ESBL phenotypes	*E. coli*, *Enterobacter* spp.	[41,45]
Multidrug	*adeF* (RND efflux pump conferring resistance to fluoroquinolones and beta-lactams)	Community DNA (Biofilm)	[34]

### Interventions and biofilm-AMR dynamics

Data regarding interventions highlighted the influence of infrastructure and chemical treatments on biofilm persistence. In terms of physical substrate, Aboelseoud et al. (2021) reported that PVC pipes in layer farms supported significantly higher bacterial densities (2*10^19 CFU/ml) compared to iron pipes (2*10^12 CFU/ml) [44]. Regarding chemical control, a longitudinal study in swine facilities found that while treatment with 0.78% peracetic acid reduced biofilm biomass and ARG loads immediately, bacterial regrowth occurred within 3–7 days [43]. Conversely, Heinemann et al. (2020) observed that specific hygiene management adaptations in a broiler farm successfully eliminated ESBL-producing bacteria from water lines, although *P. aeruginosa* persisted despite these interventions [45].

## Discussion

This systematic synthesis of ten primary studies, considered alongside broader literature on engineered-water biofilms, supports a cautious but consequential shift in how we conceptualize farm DWDS. On many farms, DWDS appear to function not as passive conduits but as engineered microhabitats that maintain surface-associated microbial communities distinct from transient planktonic inputs. Within these biofilms, microbial densities are elevated, physiological heterogeneity increases, and mobile genetic elements can accumulate [35,49]. These conditions plausibly promote the persistence and localized circulation of AMR. While current evidence does not demonstrate routine transfer of pipe-resident genes to human clinical pathogens, it identifies a credible and under-recognized reservoir that warrants explicit integration into One Health surveillance and mitigation strategies. However, we must clearly distinguish between the ecological detection of resistance genes and demonstrated epidemiological impact. Current evidence primarily establishes these systems as ecological reservoirs. Direct epidemiological pathways linking DWDS-associated resistance to human clinical infections remain theoretical and lack empirical demonstration [50].

Farm DWDS differ from municipal distribution systems in several ecological respects that favor biofilm establishment and maturation [51]. Farm water systems combine warm temperatures, narrow piping, and nutrient pulses from vaccine stabilizers or medications. This creates an environment where intermittent flow wets the surface but lacks the shear force necessary to detach accumulated biofilm [12,44,52]. In this environment, extracellular polymeric substances (EPS) form structured matrices that generate marked microscale gradients of oxygen, nutrients, and antimicrobial exposure [53]. These gradients produce stratified metabolic zones and phenotypic heterogeneity, including metabolically inactive persisters that can survive chemical shocks. The EPS itself absorbs and neutralizes oxidants, reducing the penetration depth of disinfectants that would otherwise inactivate basal cells [54–58]. High local cell densities and close physical proximity in the matrix further increase opportunities for horizontal gene transfer (HGT) through conjugation, transformation, and phage-mediated transduction [17,34,59]. Ren et al. demonstrated a significant taxonomic overlap between the oral microbiota of pigs and the biofilms established on pen water pipes, confirming that the DWDS serves as a reciprocal reservoir that is continuously re-inoculated by the host animals [34]. Collectively, these features explain why planktonic monitoring or well-head testing can substantially underestimate the microbial and genetic load actually encountered at the distal point of animal exposure, and why routine shock disinfection often yields only transient reductions in detectable organisms.

Comparisons with human premise plumbing offer a valuable perspective. In healthcare settings, the ‘last meter’ is recognized as a critical niche where pathogens persist, even when the bulk water is treated [60,61]. Applying this concept to agriculture highlights a significant monitoring gap. While protocols often test source tanks, they frequently overlook the distal nipples and troughs where animals actually drink. Consequently, farm water systems should be viewed as active biological reactors rather than passive pipes. This shift necessitates a redesign of current surveillance and mitigation strategies.

Across the included studies, there is consistent detection of biofilm-distinct microbial communities and repeated reports of multidrug-resistant isolates or resistance gene sequences in pipe-associated material [35,48]. Poultry systems in particular have shown recurrent signals of opportunistic taxa and ARGs [45,47], and at least one metagenomic study documented a diverse resistome in swine waterline coupons [43]. This resistance profile was further expanded by findings from Chinese swine farms, where metagenomic analysis identified the multidrug efflux pump gene *adeF* as the most abundant resistance determinant in stainless steel pipe biofilms [34]. This highlights that DWDS biofilms do not merely harbor transferable plasmids but also accumulate intrinsic resistance mechanisms that may complicate sanitization efforts. These converging observations across geographies and methodologies confirm that biofilms harbor significant resistance determinants.

However, the strength of inference about public-health risk and transmission pathways is constrained by methodological variation and by the limited plasmid-aware evidence. Much of the existing work relies on culture methods or targeted PCR/qPCR, which establish the presence of organisms or genes but do not by themselves demonstrate that those genes are located on mobile elements or are present in viable, transmissible plasmids [62,63]. Short-read metagenomics can identify resistance genes but often fails to determine their genomic context [64]. Consequently, confirming whether these genes are truly mobile requires long-read sequencing [65], hybrid assembly [66], or functional conjugation assays [67]. Most included studies utilized cross-sectional designs, which limits the interpretation of temporal dynamics. Consequently, it is impossible to distinguish whether ARGs persist due to active selection, historical accumulation in the infrastructure, or repeated reintroduction from the environment. These limitations do not invalidate the findings but rather clarify the boundaries of the evidence. The detection of high-priority markers, such as *mcr* and *bla*_NDM_, confirms that biofilms act as reservoirs for critical resistance traits. However, accurately quantifying the risk of transmission requires further validation through longitudinal studies and plasmid analysis.

The evidence base is also uneven across livestock sectors and geographies. Poultry predominates both because nipple-drinker systems are ubiquitous and because such enclosed, accessible systems facilitate repeated sampling. Swine and cattle, by contrast, employ a wider array of water delivery designs—bowls, troughs, bite drinkers, and wet-dry feeders—each with different flow regimes, exposure pathways, and practical sampling challenges. This sampling bias limits the broader application of current syntheses. It may mask critical variations in how water system ecology influences resistance across different livestock sectors.

If DWDS biofilms function as persistent, within-farm reservoirs of resistance determinants, then conventional approaches to water sanitation centered on planktonic indicator testing and episodic shock dosing will frequently fall short. Management strategies must shift from simple bulk-water sanitation to explicit biofilm control [35,68,69]. This requires aligning monitoring efforts with the point of animal exposure, prioritizing surface-based diagnostics over liquid sampling. The risk dynamics associated with DWDS biofilms are likely exacerbated in small-scale and backyard farming systems. These systems are particularly prevalent in low- and middle-income countries (LMICs). Unlike commercial facilities with standardized PVC piping and automated sanitation, smallholder systems frequently rely on open troughs, untreated source water, and irregular manual cleaning [70,71]. These conditions provide continuous environmental seeding and optimal conditions for biofilm maturation. From a policy perspective, mitigating this risk requires integrating DWDS management into national antimicrobial stewardship and biosecurity frameworks [72]. Policymakers should incentivize the adoption of engineered water systems designed for high-velocity flushing. Furthermore, they should promote the development of standardized biofilm sampling protocols for farm inspections and support extension programs that educate farmers on matrix-disrupting sanitation techniques [73,74]. Treatment protocols should also evolve to target the protective EPS matrix, not just the bacterial cells. For instance, enzymatic pretreatments can degrade the matrix structure, thereby increasing susceptibility to subsequent disinfectants [75–77]. From an engineering perspective, designs that minimize dead-legs and allow for high-velocity flushing are essential to reduce biomass accumulation [78].

These recommendations remain precautionary. While the biological mechanisms are plausible, the current literature lacks large-scale trials demonstrating sustained reductions in animal colonization or foodborne risk. This distinction is crucial for practical application, as interventions must prove both cost-effective and compatible with daily farm operations. Consequently, the One Health argument for managing DWDS rests on two premises. First, biofilms are proven reservoirs that sustain and redistribute resistance genes [79,80]. Second, controlling these reservoirs reduces a preventable source of exposure, even if the direct transmission risk to humans remains unquantified [81]. Therefore, treating water systems as a critical control point complements, rather than replaces, existing antimicrobial stewardship programs.

### Limitations of this synthesis and priorities for the field

The primary limitation of this review is the substantial methodological and statistical heterogeneity among the included studies, which precluded the calculation of a robust pooled estimate. A fundamental constraint is the small sample size of only ten eligible studies. This restricts our ability to extrapolate findings broadly. The ten eligible datasets employed divergent sampling protocols, ranging from non-destructive surface swabbing of drinkers [35,47] to destructive pipe-sectioning and coupon analysis [43]. These methods yield biomass metrics that are not biologically comparable. Furthermore, outcomes were bifurcated between phenotypic assays reporting MDR isolate prevalence and molecular assays quantifying resistance gene copies. Consequently, a formal meta-analysis would have lacked biological meaning, necessitating the narrative synthesis approach employed here.

Beyond synthesis constraints, the evidence base itself contains specific gaps. Geographically, the data are scattered and exhibit significant bias. The included studies are heavily concentrated in Europe and Asia, with significant voids in major livestock-producing regions in South America. This limits the global generalizability of the findings as variations in climate [82], farm infrastructure, and regional antimicrobial usage practices likely influence biofilm formation and resistome dynamics differently across the globe [82–84]. Taxonomically, poultry systems are overrepresented, likely due to the ease of accessing nipple drinker lines compared to the complex plumbing of swine and cattle facilities. Critically, there is a gap in “plasmid-aware” evidence. While studies successfully detected high-priority targets such as *mcr* and *blaNDM*, most relied on PCR or short-read metagenomics. Finally, the predominance of cross-sectional designs limits causal inference and longitudinal studies are urgently needed to distinguish between transient contamination and the active persistence of resistance traits within the biofilm matrix over successive production cycles.

In summary, the available evidence justifies prioritizing DWDS biofilms within the One Health framework for AMR. However, we must carefully distinguish between proven mechanisms and those that remain hypothetical. The ‘biofilm reactor’ concept offers a valuable model for guiding future surveillance. To translate this concept into policy, the field requires standardized methods and rigorous plasmid-level analysis. Additionally, pragmatic trials are needed to demonstrate that managing biofilms yields measurable benefits for animal and human health.

## Conclusion

This review establishes that livestock DWDS function as complex ecological reservoirs rather than passive conduits. The synthesized evidence demonstrates that biofilms within these systems sustain diverse resistomes, including critical priority pathogens and mobile genetic elements, which often evade standard disinfection regimes. Consequently, relying solely on bulk water quality monitoring underestimates the true burden of on-farm resistance. To align with One Health objectives, surveillance protocols must evolve to prioritize surface-associated sampling at the point of animal exposure. Future research should leverage long-read sequencing to map plasmid mobility within these matrices and validate scalable engineering interventions. Ultimately, integrating DWDS biofilm management into biosecurity programs offers a tangible opportunity to interrupt a recurrent cycle of antimicrobial resistance transmission at the animal-environment interface.

## Supporting information

S1 Checklist PRISMA 2020 Checklist. (DOCX)

S1 File Database-specific search strategies and syntax adaptations. Contains the exact search strings used for MEDLINE, AGRIS, PubAg, Google Scholar, and Scopus, including syntax variations for different livestock sectors.
(DOCX)

S1 Table Screening checklists. Details the specific inclusion and exclusion criteria applied during the title/abstract screening (S1 Table) and full-text eligibility assessment (S2 Table).
(DOCX)

S2 Table Consensus risk of bias assessment. Provides the domain-level risk of bias judgments (sampling, handling, laboratory, AST/genetics, and reporting) and inter-reviewer agreement for all included studies.
(DOCX)
