# Supplementary material for: The livestock drinking water system as an active reservoir for antimicrobial resistance: A systematic review and one health gap analysis
Source: PLoS One. 2026 Jun 3;21(6):e0349556. doi: 10.1371/journal.pone.0349556 (PMC13232850; doi:10.1371/journal.pone.0349556)
Supplement: S1 File — Contains the exact search strings used for MEDLINE, AGRIS, PubAg, Google Scholar, and Scopus, including syntax variations for different livestock sectors. (DOCX) [file pone.0349556.s002.docx]

# Supplementary Table S1. Database-Specific Search Strategies and Syntax Adaptations

## Table S1.1. MEDLINE (via PubMed) Search Strings

| **Scope** | **Query** |
| --- | --- |
| **Global (broad)** | (("biofilm"[MeSH Terms] OR biofilm OR "biofilm formation") AND ("drinking water"[MeSH Terms] OR "drinking water" OR "water distribution" OR "drinker" OR "nipple" OR "trough" OR "water pipe" OR "water line") AND (livestock OR poultry OR chicken OR broiler OR layer OR swine OR pig OR porcine OR cattle OR bovine OR dairy) AND ("antimicrobial resistance" OR "antibiotic resistance" OR AMR OR ARG OR "resistance gene" OR MDR)) |
| **Poultry-focused** | (("biofilm"[MeSH Terms] OR biofilm OR "biofilm formation") AND ("drinking water" OR "water distribution" OR "drinker" OR "nipple" OR "trough" OR "water line") AND (poultry OR chicken OR broiler OR layer) AND ("antimicrobial resistance" OR "antibiotic resistance" OR AMR OR ARG)) |
| **Cattle-focused** | (("biofilm"[MeSH Terms] OR biofilm OR "biofilm formation") AND ("drinking water" OR "trough" OR "water tank" OR "water distribution" OR "water line") AND (cattle OR bovine OR dairy OR beef) AND ("antimicrobial resistance" OR "antibiotic resistance" OR AMR OR ARG)) |
| **Swine-focused** | (("biofilm"[MeSH Terms] OR biofilm OR "biofilm formation") AND ("drinking water" OR "drinker" OR "nipple" OR "trough" OR "water line") AND (swine OR pig OR porcine) AND ("antimicrobial resistance" OR "antibiotic resistance" OR AMR OR ARG)) |
| **LMIC-biased** | (("biofilm"[MeSH Terms] OR biofilm) AND ("drinking water" OR "drinker" OR "nipple" OR "trough") AND (livestock OR poultry OR swine OR cattle) AND ("antimicrobial resistance" OR ARG OR "antibiotic resistance")) AND (Africa OR "South Asia" OR India OR Pakistan OR Bangladesh OR Nepal OR Nigeria OR Kenya OR Ethiopia OR "Latin America" OR Brazil OR Vietnam OR Indonesia OR "developing country" OR "low-income" OR "middle-income" OR LMIC) |

## Table S1.2. AGRIS (FAO) Search Strings

| **Scope** | **Query** |
| --- | --- |
| **Global (broad)** | ("biofilm" OR "biofilm formation") AND ("drinking water" OR "drinker" OR "nipple drinker" OR "trough" OR "water line" OR "water tank") AND (livestock OR poultry OR broiler OR layer OR swine OR pig OR porcine OR cattle OR bovine OR dairy) AND ("antimicrobial resistance" OR "antibiotic resistance" OR AMR OR ARG OR "resistance gene") |
| **Poultry-focused** | ("biofilm") AND ("drinking water" OR "drinker" OR "nipple drinker" OR "water line" OR trough) AND (poultry OR broiler OR layer OR chicken) AND ("antimicrobial resistance" OR ARG) |
| **Cattle-focused** | ("biofilm") AND ("drinking water" OR trough OR "water tank" OR "water line") AND (cattle OR bovine OR dairy OR beef) AND ("antimicrobial resistance" OR ARG) |
| **Swine-focused** | ("biofilm") AND ("drinker" OR "nipple drinker" OR "water line" OR trough) AND (swine OR pig OR porcine) AND ("antimicrobial resistance" OR ARG) |
| **LMIC-biased** | ("biofilm") AND ("drinking water" OR trough OR drinker) AND (livestock OR poultry OR swine OR cattle) AND ("antimicrobial resistance" OR ARG) AND (Africa OR "South Asia" OR India OR Pakistan OR Bangladesh OR Nepal OR Kenya OR Ethiopia OR "developing country" OR LMIC) |

## Table S1.3. PubAg (USDA) Search Strings

| **Scope** | **Query** |
| --- | --- |
| **Global (broad)** | ("biofilm" OR "biofilm formation") AND ("drinking water" OR drinker OR "nipple drinker" OR trough OR "water line" OR "water tank") AND (livestock OR poultry OR chicken OR broiler OR layer OR swine OR pig OR porcine OR cattle OR bovine OR dairy) AND ("antimicrobial resistance" OR "antibiotic resistance" OR AMR OR ARG OR "resistance gene") |
| **Poultry-focused** | ("biofilm") AND ("drinking water" OR "drinker" OR "nipple drinker" OR "water line" OR trough) AND (poultry OR broiler OR layer) AND ("antimicrobial resistance" OR ARG) |
| **Cattle-focused** | ("biofilm") AND ("drinking water" OR trough OR "water tank" OR "water line") AND (cattle OR bovine OR dairy OR beef) AND ("antimicrobial resistance" OR ARG) |
| **Swine-focused** | ("biofilm") AND ("drinker" OR "nipple drinker" OR "water line" OR trough) AND (swine OR pig OR porcine) AND ("antimicrobial resistance" OR ARG) |
| **LMIC-biased** | ("biofilm") AND ("drinking water" OR trough OR drinker) AND (livestock OR poultry OR swine OR cattle) AND ("antimicrobial resistance" OR ARG) AND (Africa OR "South Asia" OR India OR Pakistan OR Bangladesh OR Nepal OR Kenya OR Ethiopia OR "developing country" OR LMIC) |

## Table S1.4. Google Scholar Search Strings

| **Scope** | **Query** |
| --- | --- |
| **Global** | "biofilm" AND ("drinking water" OR "drinker line" OR "nipple drinker" OR "water trough" OR "water line") AND (livestock OR poultry OR broiler OR cattle OR swine) AND ("antimicrobial resistance" OR "antibiotic resistance" OR ARG) |
| **Poultry-focused** | "biofilm" AND ("nipple drinker" OR "drinker line" OR "water line" OR trough) AND (poultry OR broiler OR layer) AND ("antimicrobial resistance" OR ARG) |
| **Cattle-focused** | "biofilm" AND ("drinking water" OR trough OR "water tank" OR "water line") AND (cattle OR bovine OR dairy OR beef) AND ("antimicrobial resistance" OR ARG) |
| **Swine-focused** | "biofilm" AND ("drinker" OR "nipple drinker" OR "water line" OR trough) AND (swine OR pig OR porcine) AND ("antimicrobial resistance" OR ARG) |
| **LMIC-biased** | "biofilm" AND ("drinking water" OR trough OR drinker) AND (livestock OR poultry OR swine OR cattle) AND ("antimicrobial resistance" OR ARG) AND (Nepal OR India OR Pakistan OR "South Asia" OR Africa OR Kenya OR Ethiopia OR "developing country" OR LMIC) |

## Table S1.5. Scopus (Elsevier) Search Strings

| **Scope** | **Query** |
| --- | --- |
| **Global (master)** | TITLE-ABS-KEY(biofilm*) AND TITLE-ABS-KEY("drinking water" OR "water distribution" OR "drinker line" OR "nipple drinker" OR "water trough" OR "water line" OR "water pipe" OR "water tank") AND TITLE-ABS-KEY(livestock OR poultry OR chicken* OR broiler* OR layer* OR swine OR pig* OR porcine OR cattle OR bovine OR dairy OR ruminant*) AND TITLE-ABS-KEY("antimicrobial resistan*" OR "antibiotic resistan*" OR "resistance gene*" OR ARG* OR AMR OR MDR) |
| **Poultry-focused** | TITLE-ABS-KEY(biofilm*) AND TITLE-ABS-KEY("drinking water" OR "drinker line" OR "nipple drinker" OR "water line" OR "water trough") AND TITLE-ABS-KEY(poultry OR chicken* OR broiler* OR layer*) AND TITLE-ABS-KEY("antimicrobial resistan*" OR "antibiotic resistan*" OR "resistance gene*" OR ARG* OR AMR) |
| **Cattle-focused** | TITLE-ABS-KEY(biofilm*) AND TITLE-ABS-KEY("drinking water" OR "water trough" OR "water tank" OR "water line" OR "water distribution") AND TITLE-ABS-KEY(cattle OR bovine OR dairy OR beef OR calf OR calves) AND TITLE-ABS-KEY("antimicrobial resistan*" OR "antibiotic resistan*" OR "resistance gene*" OR ARG* OR AMR) |
| **Swine-focused** | TITLE-ABS-KEY(biofilm*) AND TITLE-ABS-KEY("drinking water" OR "nipple drinker" OR "water line" OR "water trough" OR "water tank") AND TITLE-ABS-KEY(swine OR pig* OR porcine OR sow OR piglet*) AND TITLE-ABS-KEY("antimicrobial resistan*" OR "antibiotic resistan*" OR "resistance gene*" OR ARG* OR AMR) |
| **LMIC-focused** | TITLE-ABS-KEY(biofilm*) AND TITLE-ABS-KEY("drinking water" OR "water line" OR "water trough" OR "nipple drinker") AND TITLE-ABS-KEY(livestock OR poultry OR swine OR cattle) AND TITLE-ABS-KEY("antimicrobial resistan*" OR "antibiotic resistan*" OR "resistance gene*" OR ARG* OR AMR) AND TITLE-ABS-KEY(Africa OR "South Asia" OR India OR Pakistan OR Bangladesh OR Nepal OR Nigeria OR Kenya OR Ethiopia OR "Latin America" OR Brazil OR Vietnam OR Indonesia OR "developing countr*" OR "low income" OR "middle income") |
