# Supplementary material for: The livestock drinking water system as an active reservoir for antimicrobial resistance: A systematic review and one health gap analysis
Source: PLoS One. 2026 Jun 3;21(6):e0349556. doi: 10.1371/journal.pone.0349556 (PMC13232850; doi:10.1371/journal.pone.0349556)
Supplement: S1 Table — Details the specific inclusion and exclusion criteria applied during the title/abstract screening (S1 Table) and full-text eligibility assessment (S2 Table). (DOCX) [file pone.0349556.s003.docx]

# Supplementary Table S2. Title and Abstract Screening Checklist

*This checklist was used to filter records during the first phase. If the answer to any "Inclusion" question was "Yes" or "Unclear," the record was retained for full-text review. If the answer to any "Exclusion" question was "Yes," the record was excluded.*

| **Screening Question** | **Criteria Type** | **Decision Rule** |
| --- | --- | --- |
| **1. Is the study about livestock?** (Poultry, cattle, swine, small ruminants) | Inclusion | If **No**: Exclude |
| **2. Does the study mention drinking water systems?** (DWDS, troughs, pipes, water lines, etc.) | Inclusion | If **No:** Exclude |
| **3. Does the study mention antimicrobial resistance (AMR), residues, or biofilms?** | Inclusion | If **No:** Exclude |
| **4. Is the article a review, commentary, or editorial without primary data?** | Exclusion | If **Yes:** Exclude |

# Supplementary Table S3. Full-Text Eligibility and Exclusion Checklist

*This detailed checklist was applied to all retrieved full-text articles. A study must meet* ***all*** *inclusion conditions to be accepted. The primary reason for exclusion was recorded for the PRISMA flow diagram.*

| **Category** | **Detailed Criteria** | **Action if unmet** |
| --- | --- | --- |
| **1. Population** | **Include:** Commercial or smallholder poultry, cattle, swine, or small ruminants.  **Exclude:** Humans, wildlife, aquaculture, companion animals. | **Exclude Code 1:** Wrong Population |
| **2. Sample Source** | **Include:** Biofilm material sampled directly from farm DWDS (pipes, nipples, tanks, coupons).  **Exclude:** Bulk water only, soil, manure, or laboratory-only models without farm validation. | **Exclude Code 2:** Wrong Sample/Setting |
| **3. Outcome** | **Include:** Reports at least one of: (a) Phenotypic AST of biofilm, (b) ARG detection/quantification in biofilm, or (c) Antimicrobial residues in biofilm.  **Exclude:** Planktonic data only; no AMR data. | **Exclude Code 3:** Wrong Outcome |
| **4. Study Type** | **Include:** Primary empirical research.  **Exclude:** Reviews, conference abstracts (if insufficient data), simulation studies. | **Exclude Code 4:** Wrong Study Design |
