# Supplementary material for: The livestock drinking water system as an active reservoir for antimicrobial resistance: A systematic review and one health gap analysis
Source: PLoS One. 2026 Jun 3;21(6):e0349556. doi: 10.1371/journal.pone.0349556 (PMC13232850; doi:10.1371/journal.pone.0349556)
Supplement: S2 Table — Provides the domain-level risk of bias judgments (sampling, handling, laboratory, AST/genetics, and reporting) and inter-reviewer agreement for all included studies. (DOCX) [file pone.0349556.s004.docx]

# Supplementary Table S3. Consensus Risk of Bias Assessment for Included Studies (n = 10)

## **Table S3.1 — Domain-level and overall risk-of-bias judgments**

| **Study ID** | **Sampling** | **Handling** | **Laboratory**  **QC** | **AST / Genetics** | **Reporting** | **Overall**  **RoB** |
| --- | --- | --- | --- | --- | --- | --- |
| Doughan et al. (2025) | Low | Low | Moderate | Moderate | Low | Low |
| Piccirillo et al. (2024) | Low | Low | Low | Low | Low | Low |
| Vougat Ngom et al. (2025) | Low | Low | Low | Low | Low | Low |
| Grudlewska-Buda et al. (2023) | Moderate | Moderate | Moderate | Low | Moderate | Moderate |
| Hayer et al. (2022) | Low | Low | Low | Moderate | Low | Low |
| Aboelseoud et al. (2021) | Moderate | Moderate | High | Moderate | Moderate | Moderate |
| Ahangaran et al. (2022) | Moderate | Low | Moderate | Moderate | Moderate | Moderate |
| Grakh et al. (2022) | Moderate | Moderate | Moderate | Moderate | Moderate | Moderate |
| Heinemann et al. (2020) | High | Low | Low | Low | Moderate | Moderate |
| Ren et al. (2025) | Low | Low | Low | Low | Low | Low |

*Low = low risk of bias for that domain (methods/reporting adequate). Moderate = some limitations that may affect validity but not sufficient to invalidate results. High = important limitations likely to bias findings. NA = domain not applicable (e.g., molecular QC NA where no molecular work was performed).*

Judgments were based solely on the study metadata provided to the review; full study reports may provide additional information that could change ratings.

## **Table S3.2 — Inter-reviewer overall risk-of-bias agreement**

| **Study ID** | **Reviewer 1 (Overall RoB)** | **Reviewer 2 (Overall RoB)** | **Agreement?** |
| --- | --- | --- | --- |
| Doughan et al. (2025) | Moderate | Low | No |
| Piccirillo et al. (2024) | Low | Low | Yes |
| Vougat Ngom et al. (2025) | Low | Low | Yes |
| Grudlewska-Buda et al. (2023) | Moderate | Moderate | Yes |
| Hayer et al. (2022) | Moderate | Low | No |
| Aboelseoud et al. (2021) | Moderate | Moderate | Yes |
| Ahangaran et al. (2022) | Moderate | Moderate | Yes |
| Grakh et al. (2022) | Moderate | Moderate | Yes |
| Heinemann et al. (2020) | Low | Moderate | No |
| Ren et al. (2025) | Low | Low | Yes |

# Risk of Bias (RoB) Rubric

Each included study was evaluated across five domains: Sampling, Sample Handling, Laboratory Quality Control (QC), AST/Genetics Methodology, and Reporting Transparency. Domains were rated as Low, Moderate, or High risk of bias according to the criteria below. Judgments were based solely on the information reported within each study.

## **Sampling (Selection Bias)**

Low: Random or systematic selection; multiple farms/sites; clear criteria; adequate sample size.

Moderate: Convenience sampling; single-region without justification; partial description of selection.

High: Pre-selected problem farms; outbreak-only sampling; no description of selection.

1. **Sample Handling (Pre-analytical Bias)**

Low: Controlled temperature; defined transport time; sterile technique; standardized handling.

Moderate: Partial handling information; minor contamination/degradation risk.

High: No handling description; high likelihood of contamination or degradation.

1. **Laboratory Quality Control (Methodological Reliability)**

Low: Internal/external controls; replicates; sequencing QC metrics; plate controls.

Moderate: Some QC elements missing or incompletely described.

High: No QC reported; unvalidated methods; high likelihood of artefacts.

1. **AST / Genetics Methodology (Measurement Bias)**

Low: CLSI/EUCAST standards; validated PCR or sequencing workflows; appropriate reference databases.

Moderate: Limited gene panels; partial validation; deviations from standards.

High: Non-validated assays; no standards cited; unclear gene identification.

1. **Reporting Transparency (Reporting Bias)**

Low: Complete methods; full outcome reporting; supplementary data provided; limitations discussed.

Moderate: Incomplete tables/figures; partial transparency.

High: Selective reporting; missing outcomes; ambiguous results.

**Overall Risk-of-Bias Judgment**

• Low RoB: All domains Low or only one Moderate.

• Moderate RoB: Two or more domains Moderate OR one High.

• High RoB: Multiple High domains OR critical bias in Sampling or Lab QC.

**Inter-reviewer Procedure**

Two reviewers independently assessed risk of bias. Discrepancies were resolved by consensus discussion. Agreement is reported as Yes/No in Table B.
